# Repeated Abscopal Effect After Two Courses of Stereotactic Body Radiotherapy for Multiple Postoperative Metastases of Malignant Melanoma of the Buccal Mucosa

**DOI:** 10.7759/cureus.103571

**Published:** 2026-02-13

**Authors:** Masahiro Kawahara, Yukiko Fukuda, Keiko Akahane, Machi Nakagawa, Masashi Endo, Kazunari Ogawa, Satoru Takahashi, Michiko Nakamura, Daisuke Irie, Hideki Watanabe, Noriko Oyama-Manabe, Katsuyuki Shirai

**Affiliations:** 1 Department of Radiology, Jichi Medical University Saitama Medical Center, Saitama, JPN; 2 Department of Radiology, Jichi Medical University Hospital, Shimotsuke, JPN; 3 Department of Dentistry, Oral and Maxillofacial Surgery, Jichi Medical University Saitama Medical Center, Saitama, JPN

**Keywords:** abscopal effect, immune checkpoint inhibitors, malignant melanoma, metastatic lung tumor, stereotactic body radiotherapy

## Abstract

We report a case of repeated abscopal effect (AE) following two courses of stereotactic body radiotherapy (SBRT) for multiple postoperative metastases of malignant melanoma in the buccal mucosa during treatment with nivolumab. A 90-year-old female presented with malignant melanoma of the right buccal mucosa. She underwent partial resection of the right buccal mucosa and right cervical dissection at our hospital in January 2022. In August 2022, positron emission tomography (PET)-CT revealed recurrence in the left cervical lymph node and metastatic lung tumors at S5 of the left lung and S9 of the right lung. SBRT (40 Gy in five fractions) was performed for the recurrent left cervical lymph node. In December 2022, PET-CT showed regression of the irradiated lymph nodes as well as an unirradiated left lung S5 lesion, which was considered the first AE. In addition to the existing S9 lesion in the right lung, a new metastatic lung tumor was identified in S4 of the right lung, and SBRT of 42 Gy in four fractions was performed on the right S9 lesion in April 2023. In July 2023, PET-CT showed regression of both the irradiated right S9 lesion and the unirradiated right S4 lesion, representing the second AE. Both the irradiated and unirradiated lesions associated with AE remained reduced on PET-CT in October 2023. Repeated AEs after two SBRT courses during nivolumab therapy are extremely rare. We report this case together with a literature review.

## Introduction

The abscopal effect (AE) is a rare phenomenon in which metastatic lesions regress at sites distant from the irradiated area. Typically, radiotherapy affects only the targeted region and induces regression of the treated disease; however, activation of the immune system can produce anticancer effects on lesions outside the irradiated field. Consequently, the AE is particularly pronounced when radiotherapy is combined with immune checkpoint inhibitors (ICIs). Recent radiotherapy-immuno-oncology studies have highlighted the potential synergy between radiotherapy and ICIs, such as nivolumab, in enhancing abscopal responses [1,2]. Although numerous studies on the AE have been reported over the years, it remains a rare phenomenon in clinical practice.

Oral and buccal mucosal melanoma is a rare, highly malignant tumor, accounting for less than 2% of all melanomas. It is associated with a poor prognosis due to delayed diagnosis and limited treatment options. Standard treatment typically involves surgical resection when feasible, combined with adjuvant radiotherapy or systemic therapy; however, treatment outcomes remain suboptimal. Compared with cutaneous melanoma, mucosal melanoma is biologically distinct and usually detected at a more advanced stage because primary sites are often occult. It is associated with poorer survival and a different mutational spectrum, with a lower frequency of BRAF V600 mutations and relatively higher rates of KIT and NRAS alterations. These distinctions are clinically relevant when considering systemic therapy and the likelihood of observing abscopal phenomena [3-5].

In this study, we observed repeated AEs following two courses of stereotactic body radiotherapy (SBRT) during treatment with nivolumab in a patient with malignant melanoma of the buccal mucosa with multiple metastases. This case is notable for several reasons: (1) two distinct abscopal responses were observed after SBRT, (2) the responses occurred in different non-irradiated lesions, and (3) the patient was very elderly, making systemic therapy particularly challenging. This case provides valuable insight, as there have been few reports documenting the occurrence of two AEs in the same patient. By presenting detailed imaging, treatment timelines, and clinical outcomes, we aim to contribute to the understanding of this rare phenomenon and its potential implications for future therapeutic strategies.

## Case presentation

**Figure 1:**
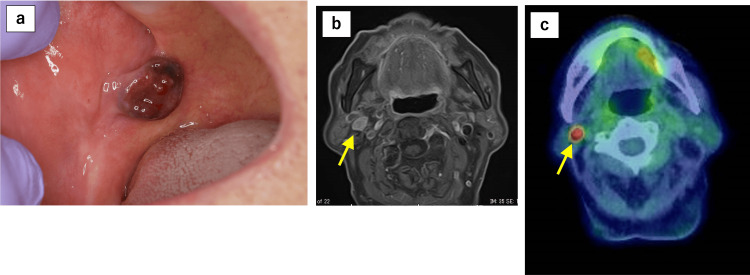
Initial imaging findings of buccal mucosal malignant melanoma and cervical lymph node metastasis (a) A polypoid mass is observed on the right buccal mucosa, and buccal mucosal malignant melanoma is diagnosed by biopsy. Right cervical lymph node metastasis is detected on (b) MRI (gadolinium-enhanced T1-weighted imaging with fat suppression) and (c) PET-CT MRI: magnetic resonance imaging; PET-CT: positron emission tomography-computed tomography

A 90-year-old female presented with a dark purple mass in the right buccal mucosa and reported frequent accidental biting of the lesion. The patient had earlier visited a local dentist, where a polypoid mass had been observed in the right buccal mucosa, and she had been referred to our hospital for further examination and treatment. Her relevant comorbidities included hypertension, dyslipidemia, glaucoma, and age-related macular degeneration. The patient’s performance status was 1 at the time of presentation. A biopsy was performed, and the patient was diagnosed with buccal mucosal malignant melanoma. Contrast-enhanced CT, MRI, and positron emission tomography-CT (PET-CT) demonstrated metastasis to the right cervical lymph nodes, resulting in a clinical stage of cT3N1M0 (Figure 1).

**Figure 2:**
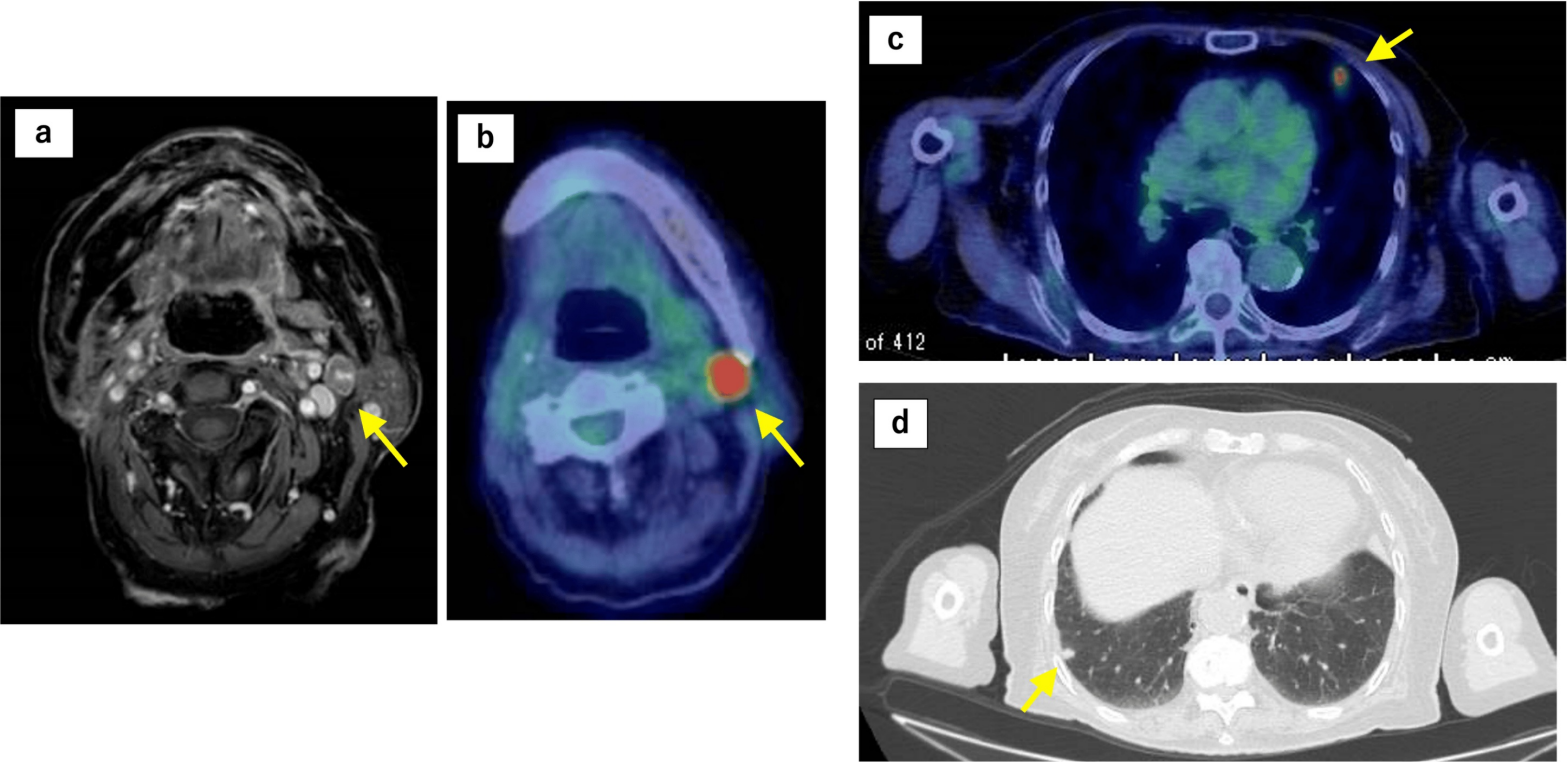
Baseline imaging after initial surgery prior to first SBRT PET-CT images. (a and b) Recurrence of left cervical lymph nodes, (c) metastatic lung tumor in left lung S5, and (d) right lung S9 SBRT: stereotactic body radiotherapy; PET-CT: positron emission tomography-computed tomography

In January 2022, a partial resection of the right buccal mucosa and right cervical lymph node dissection were performed. Intraepithelial spread of atypical melanocytes extending up to 25 mm was observed, and the patient was diagnosed with malignant melanoma classified as pT3. The horizontal and vertical margins were confirmed to be negative. The lymph nodes were evaluated according to anatomical lymph node levels: IB (0/2), IIA (2/2), IIB (1/3), III (0/1), resulting in pN1 (3/8). One month after surgery, nivolumab (240 mg) was administered every two weeks. In August 2022, after 12 cycles of nivolumab, cervical MRI revealed metastasis to the left cervical lymph nodes. PET-CT also revealed a left cervical lymph node lesion and metastatic lung tumors in the left S5 and right S9 segments (Figure 2). The left cervical lymph node was considered inoperable because of adhesion to the left internal jugular vein. Although nivolumab treatment was continued, radiotherapy was planned for all three lesions.

**Figure 3:**
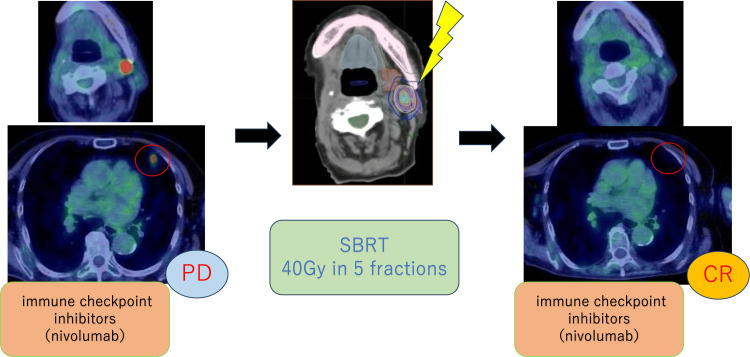
First abscopal effect observed after SBRT to cervical lymph node metastasis PET-CT images of the first abscopal effect. Three months after SBRT was performed for the left cervical lymph node metastasis, the metastatic lesion in the left lung S5 disappeared on PET-CT SBRT: stereotactic body radiotherapy; PET-CT: positron emission tomography-computed tomography

**Figure 4:**
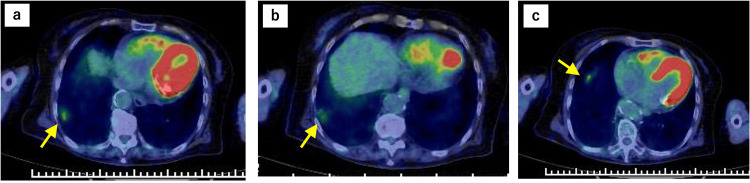
Pre-second SBRT imaging showing new pulmonary metastatic lesions (a, b) PET-CT showed new lesions in the vicinity of the S9 lesion in the right lung and (c) a new metastatic lesion in the S4 of the right lung SBRT: stereotactic body radiotherapy; PET-CT: positron emission tomography-computed tomography

To avoid the risk of interstitial pneumonia from immune-related adverse events (irAEs) associated with nivolumab, stereotactic body radiotherapy (SBRT) to the lungs was minimized. Therefore, SBRT was prioritized for the left cervical lymph node, and metastatic lung lesions were monitored for the time being. In September 2022, SBRT of 40 Gy in five fractions was performed for left cervical lymph node metastasis. Nivolumab continued to be administered biweekly during irradiation. Three months after treatment, PET-CT showed regression of the irradiated left cervical lymph node lesion and regression of the left lung S5 lesion outside the irradiation field. This regression of the unirradiated lesion was considered the first AE (Figure 3). The mean dose to S5 was 0.01 Gy (Dmax 0.01 Gy). The S5 lesion decreased from 12.49 mm to undetectable, and SUVmax dropped from 5.35 to 0, meeting RECIST criteria for complete response. In March 2023, PET-CT revealed hypermetabolism near the existing right lung S9 lesion and a new hypermetabolic lesion in the right lung S4. Three metastatic lung lesions were identified, and our department recommended radiotherapy (Figure 4).

**Figure 5:**
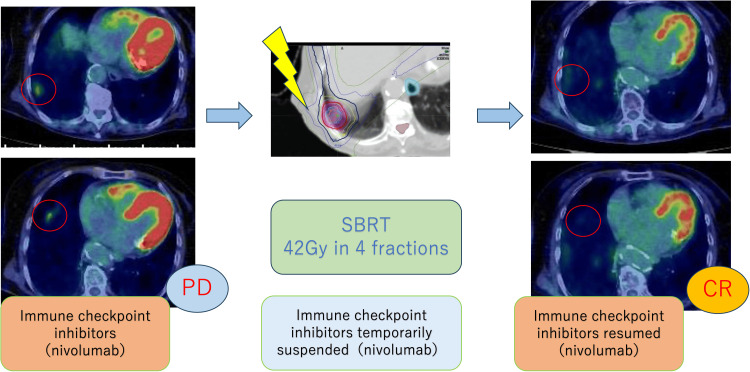
Second abscopal effect observed after SBRT to right lung S9 lesion PET-CT images of the second abscopal effect. Four months after SBRT was performed on the right lung S9 lesion, the metastatic lesion in the right lung S4 disappeared on PET-CT, indicating an abscopal effect SBRT: stereotactic body radiotherapy; PET-CT: positron emission tomography-computed tomography

Among the three lesions, two metastases in the right lung S9 were close to each other and could be treated in a single SBRT session. To avoid the risk of interstitial pneumonia related to irAEs, we decided to leave a six-month interval before performing SBRT on the right lung S4 lesion. Nivolumab was held for one dose to reduce the risk of irAEs, with a one-week rest period both before and after irradiation. In April 2023, a second SBRT of 42 Gy in four fractions was performed on the two right lung S9 metastatic lesions. This SBRT was delivered within safe dose constraints, achieving Lungs V20Gy of 7.6%, Lungs V5Gy of 19.9%, and a mean lung dose of 4.60 Gy. Four months after treatment, PET-CT revealed regression of the irradiated right lung S9 lesions and regression of the unirradiated right lung S4 lesion. This was considered the second AE, as the lesion that was not irradiated also regressed (Figure 5).

**Figure 6:**
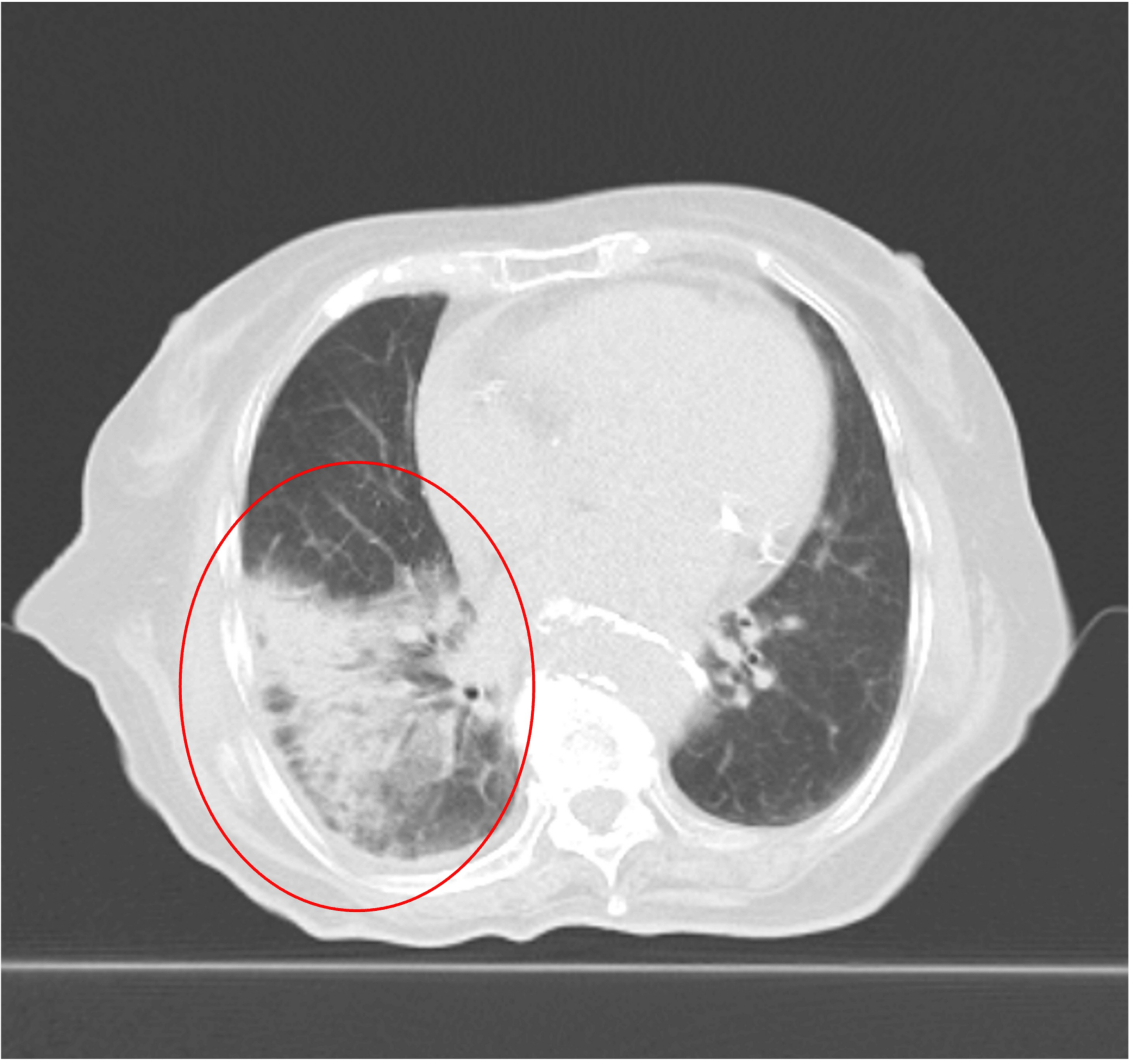
CT imaging four months after SBRT showing extensive pulmonary infiltrates CT 4 months after SBRT showed wide infiltration shadows inside and outside the irradiation range SBRT: stereotactic body radiotherapy; CT: computed tomography

**Figure 7:**
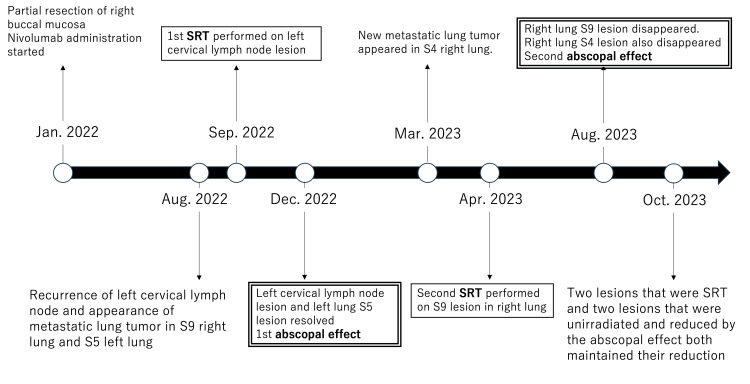
Timeline from the start of treatment to the occurrence of two abscopal effects

The mean dose to S4 was 7.78 Gy (Dmax 9.65 Gy). The S4 lesion decreased from 12.3 mm to undetectable, and SUVmax dropped from 2.690 to 0, meeting RECIST criteria for complete response. Four months after the second SBRT session, CT showed extensive pulmonary infiltrative shadows inside and outside the irradiated area, which were considered pneumonitis because of radiotherapy and irAE (Figure 6). Pneumonitis was classified as CTCAE Grade 2, occurring approximately 16 weeks after SBRT. Nivolumab was discontinued, and prednisolone was started. PET-CT in October 2023 showed that the two irradiated lesions and the two lesions associated with the AE had shrunk. The timeline from the start of treatment to the two AEs is shown in Figure 7.

## Discussion

AE is a phenomenon in which antitumor effects occur in non-irradiated areas after irradiating a local site, resulting in a reduction of lesions distant from the irradiated area. Activation of the immune system plays an important role in this phenomenon. There are numerous reports describing the AE [6-9]. However, our research has revealed very few reports of multiple AEs occurring in a single patient, as observed in this case. This case was evaluated from several perspectives. Alternative explanations for the regression of non-irradiated lesions should be considered. Because the patient continued nivolumab throughout the clinical course, a delayed or heterogeneous response to immune checkpoint inhibition cannot be completely excluded. In addition, spontaneous regression, although extremely rare in malignant melanoma, represents another theoretical possibility. Although these competing explanations were explicitly considered in our analysis, the reproducible temporal association with SBRT and the spatially distinct regression patterns make these alternatives less likely.

However, several observations support an abscopal effect interpretation. First, in both episodes, regression of the non-irradiated lesions occurred within a defined temporal window following SBRT, suggesting a radiation-triggered systemic effect rather than a gradual immunotherapy-driven response. Second, each abscopal response was accompanied by local control of the irradiated lesion, followed by synchronous regression of a spatially distinct, non-irradiated metastasis. Third, the phenomenon occurred twice under continued nivolumab therapy, each time after SBRT to a different target lesion, arguing against a random or purely systemic effect and supporting a reproducible radiation-associated immune mechanism.

Taken together, while alternative explanations cannot be completely excluded in a single-case report, the consistent temporal relationship between SBRT and distant tumor regression supports an abscopal mechanism as the most plausible explanation in this case. The occurrence of two sequential AEs is rare. Zhang et al. reported two AEs in patients with renal cell carcinoma [10]. In that report, the patient had previously received pembrolizumab and axitinib, and after SBRT for a retroperitoneal mass, the first AE was observed in the subcutaneous abdominal wall and splenic hilar mass. After SBRT for a metastatic pelvic lesion, a second AE was observed in the same two sites. Although the two AEs were observed at the same sites in that report, in our case, they occurred at different sites after two separate courses of SBRT. This is the first reported case of sequential AEs occurring at distinct sites and will provide valuable insights for the future development of combined radiotherapy and immune checkpoint inhibitor (ICI) therapy.

In this case, the primary disease was buccal mucosal malignant melanoma, which is rare among malignant melanomas. Chang et al. reported that the distribution of melanoma by primary site was 91.2% in the skin, 5.2% in the eye, 1.3% in the mucosa, and 2.2% unknown [11]. Among mucosal malignant melanomas, the site distribution was 55.4% in the head and neck, 18.0% in the female genital tract, 23.8% in the anus/rectum, and 2.8% in the urinary tract. Sohal et al. reported that mucosal malignant melanoma is rare, has a poor prognosis, and is often diagnosed late due to its hidden occurrence on various mucosal surfaces. Radiotherapy and combination immunotherapy (nivolumab + ipilimumab) have been shown to be safe and effective [12]. Several cases of AEs in cutaneous malignant melanoma have been reported; however, only a few studies have documented AEs in oral mucosal malignant melanoma. Igarashi et al. reported AEs in a patient with malignant melanoma of the left hard palate [13]. That patient underwent maxillectomy, total neck dissection, and postoperative adjuvant nivolumab therapy. The patient had multiple brain, splenic, and hepatic metastases, and regression of the splenic and hepatic metastases was observed after whole-brain irradiation with 30 Gy in 10 fractions.

Tsui et al. reported an AE in malignant melanoma that spread from the right buccal mucosa to the soft and hard palates [14]. After partial resection of the right maxilla and marginal resection of the mandible with a free vascularized flap, adjuvant radiotherapy of 50 Gy in 20 fractions was delivered to the resected primary tumor site. Follow-up MRI revealed recurrent lesions on the floor of the mouth outside the irradiation field and multiple pulmonary nodules in the thoracic cavity. Epacadostat + pembrolizumab was initiated; however, the cervical tumor continued to enlarge. Palliative irradiation of 24 Gy in three fractions on days 0, 7, and 21 was delivered to the cervical tumor using intensity-modulated radiation therapy. After irradiation, regression of the cervical mass was observed, along with simultaneous regression of multiple pulmonary metastases. The AE in primary mucosal malignant melanoma is rare, and our case is particularly valuable because it was observed twice at distinct sites (Table 1).

**Table 1:** Comparison of radiation parameters and systemic therapy context with previously reported cases This table summarizes radiation prescriptions, techniques, and the systemic therapy context temporally associated with documented AE in two published reports and in the present case AE: abscopal effect; SBRT: stereotactic body radiotherapy; IMRT: intensity modulated radiotherapy

Reference	AE‑triggering irradiation (site)	Prescription/fractionation	Technique	Concomitant/ongoing systemic therapy	Notes on AE pattern
Zhang et al. [10]	RT#1: retroperitoneal mass → RT#2: pelvic metastasis	30 Gy/6 fr (SBRT) ×2 courses	SBRT	Prior pembrolizumab (stopped due to financial issues)	Two abscopal events at the same two distant sites
Igarashi et al. [13]	Whole brain	30 Gy/10 fr	Conventional external‑beam RT	Nivolumab (adjuvant/ongoing)	AE in the spleen and liver
Tsui et al. [14]	Cervical tumor	24 Gy/3 fr (days 0, 7, 21)	IMRT	Epacadostat + pembrolizumab	AE in neck lesion and multiple lung metastases
Present case – SBRT #1	Left cervical LN (Sep 2022)	40 Gy/5 fr	SBRT	Nivolumab continued	1st AE: left lung S5 → CR
Present case – SBRT #2	Right lung S9 (two lesions) (Apr 2023)	42 Gy/4 fr	SBRT	Nivolumab with a brief pause (±1 week around RT)	2nd AE: right lung S4 → CR

In the present case, extensive pulmonary fibrosis developed unexpectedly after the second SBRT session. Although the combination of SBRT and ICIs is a promising therapeutic strategy, the risk of pneumonitis may be higher than with SBRT alone. Several reports have indicated an increased risk of radiation-induced pneumonitis when SBRT is combined with ICIs. Tian et al., in their multicenter safety and toxicity analysis, demonstrated that the combination of SBRT and ICIs significantly increased the risk of grade 3 pneumonitis compared to SBRT alone (10.7% vs. 0%, p<0.01), whereas the incidence of any-grade pneumonitis was similar (33.9% vs. 27.9%, p=0.47) [15]. Notably, the risk of any-grade pneumonitis was markedly elevated with ICI/ICI combination therapy (62.5% vs. 29.2%). Fortunately, our patient did not experience significant respiratory symptoms. However, careful assessment of pneumonitis risk is essential when SBRT is combined with ICIs.

This report has important limitations. As a single-case observation, causal inference is inherently limited. Although temporal reproducibility after each SBRT course strongly supports an abscopal effect, the possibility of a delayed or heterogeneous response to ongoing nivolumab therapy cannot be definitively excluded. Therefore, the present case should be interpreted as providing suggestive rather than conclusive evidence of repeated abscopal responses.

## Conclusions

We encountered an extremely rare phenomenon in which the AE occurred twice after two courses of SBRT for mucosal malignant melanoma. Objective measurements confirmed a complete response in non-irradiated lesions, supporting the plausibility of sequential AEs. However, alternative explanations, such as a delayed response to immunotherapy, cannot be excluded, and further research is needed to validate these observations. The AE of SBRT in combination with ICIs may represent a promising strategy to improve clinical outcomes.
